# Circulating Levels of Calprotectin as a Biomarker in Patients With Coronary Artery Disease: A Systematic Review and Meta‐Analysis

**DOI:** 10.1002/clc.24315

**Published:** 2024-07-04

**Authors:** Tara Reshadmanesh, Amir Hossein Behnoush, Maedeh Farajollahi, Amirmohammad Khalaji, Elina Ghondaghsaz, Hassan Ahangar

**Affiliations:** ^1^ School of Medicine Zanjan University of Medical Science Zanjan Iran; ^2^ School of Medicine Tehran University of Medical Sciences Tehran Iran; ^3^ Non‐Communicable Diseases Research Center, Endocrinology and Metabolism Population Sciences Institute Tehran University of Medical Sciences Tehran Iran; ^4^ School of Medicine Isfahan University of Medical Sciences Isfahan Iran; ^5^ Undergraduate Program in Neuroscience University of British Columbia Vancouver British Columbia Canada; ^6^ Department of Cardiology, School of Medicine, Mousavi Hospital Zanjan University of Medical Sciences Zanjan Iran

**Keywords:** acute coronary syndrome, calprotectin, coronary artery disease, meta‐analysis, systematic review

## Abstract

**Background:**

Calprotectin, also known as MRP8/14, is generated by immune cells and is altered in several inflammatory diseases. Studies have assessed their levels in patients with coronary artery disease (CAD) and its subtypes (stable CAD and acute coronary syndrome [ACS]). Herein, we aimed to systematically investigate these associations through a systematic review and meta‐analysis.

**Methods:**

A systematic search was conducted in four online databases, including PubMed, Scopus, Embase, and the Web of Science. Relevant studies were retrieved, screened, and extracted. Random‐effect meta‐analysis was performed for the calculation of standardized mean difference (SMD) and 95% confidence interval (CI). Blood calprotectin levels were compared between CAD patients and controls, as well as CAD subtypes.

**Results:**

A total of 20 studies were included in the systematic review and meta‐analysis, comprising 3300 CAD patients and 1230 controls. Patients with CAD had significantly higher calprotectin levels (SMD 0.81, 95% CI 0.32−1.30, *p* < 0.01). Similarly, patients with ACS were reported to have higher levels compared to those with stable CAD. However, there was no significant difference in terms of blood calprotectin levels between stable CAD cases and healthy controls. Finally, studies have shown that calprotectin could be used as a diagnostic biomarker of CAD while also predicting major adverse events and mortality in these patients.

**Conclusion:**

Based on our findings, calprotectin, as an inflammatory marker, could be used as a possible biomarker for patients with CAD and ACS. These suggest the possibility of pathophysiological pathways for this involvement and warrant further research on these associations as well as their clinical utility.

## Graphical abstract

The summary of search, included studies, and main findings of the systematic review and meta‐analysis of calprotectin levels in patients with coronary artery disease is discussed.

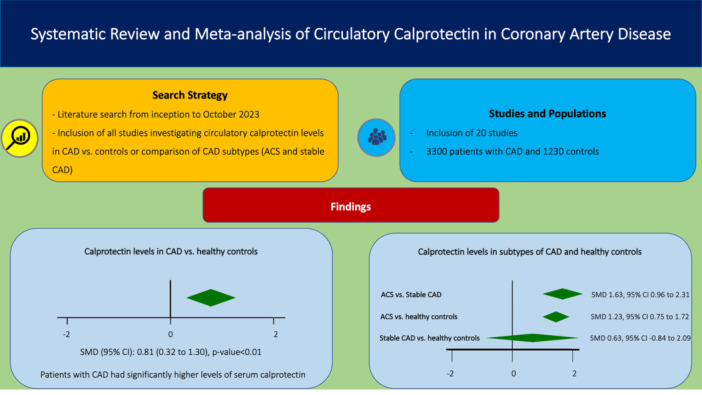

## 1 Introduction

Coronary artery disease (CAD) is characterized by atherosclerosis in coronary arteries [1], and CAD‐related mortality has been predicted to increase through 2030 [1, 2]. This condition puts a substantial medical and economic burden on families and healthcare systems [3, 4, 5]. Despite the improvement in the prognosis of CAD patients, the prevalence of CAD remains high due to worsening cardiovascular risk factors [6, 7, 8]. Due to the silent nature of atherosclerosis progression over decades, it is essential to identify high‐risk patients without further delays to prevent severe cardiovascular events since the prevention of fatal consequences of acute coronary syndrome (ACS) needs prompt evaluation and early risk stratification of these individuals [9, 10]. Several diagnostic tests are used to diagnose ACS and CAD, including electrocardiogram (ECG), cardiac biomarkers including troponin and creatine kinase myocardial band (CK‐MB), and coronary artery angiography [11, 12, 13]. However, because ACS is caused by atherosclerotic plaques that result from a chronic inflammatory condition [14], biomarkers of plaque inflammation may present more precise diagnostic tools and prognostic information.

Calprotectin, also known as MRP8/MRP14 or calgranulin A/calgranulin B, is a non‐covalently linked dimer of the S100A8 and S100A9 calcium‐binding proteins and can also exist in a tetrameric form. As an acute phase reactant, calprotectin is predominantly generated by immune cells, including those that play a vital role in acute atherosclerotic events (neutrophils, monocytes, and platelets) [15, 16, 17]. The diagnostic and prognostic significance of calprotectin as a biomarker has been assessed in multiple inflammatory disorders [18, 19, 20]. Regarding cardiovascular conditions, elevated calprotectin levels have been reported in individuals with CAD and ACS, suggesting a significant role of this biomarker in the development of cardiovascular events [21, 22]. Some studies have explored its diagnostic and prognostic potential, along with the possibility of targeting this complex in the treatment of ACS [22, 23]. In this systematic review and meta‐analysis, we addressed the lack of a pooled estimate examining the link between circulating calprotectin and ACS. Our objective was to investigate differences in calprotectin levels in patients with ACS, or stable CAD, versus those without known cardiovascular disease. Moreover, we explored the diagnostic and prognostic implications of calprotectin in patients with ACS to provide a more comprehensive understanding of the clinical relevance and utility of this biomarker.

## 2 Methods

### 2.1 Study Protocol

This systematic review and meta‐analysis was conducted and reported based on the Preferred Reporting Items for Systematic Reviews and Meta‐Analyses (PRISMA) 2020 recommendations [24]. The protocol of this study has been registered on PROSPERO (International Prospective Register of Systematic Reviews) as CRD42023454374.

### 2.2 Search Strategy

Four online databases (Web of Science, PubMed, Scopus, and Embase) were systematically searched using pre‐defined keywords on October 16, 2023. We used keywords related to CAD (e.g., “coronary,” “ischemic heart,” “acute coronary syndrome,” “ischemic heart,” “coronary artery disease,” or “myocardial ischemia”) and calprotectin (e.g., “calprotectin,” “calgranulin,” “S100A8 and A9,” or “leukocyte L1 protein”). Moreover, appropriate MeSH (Medical Subject Headings) terms were added to the PubMed database query. The exact search queries and number of search results with each query are available in Supporting Information S1: Table 1.

### 2.3 Inclusion and Exclusion Criteria

Inclusion criteria were studies evaluating (1) serum/plasma calprotectin levels in patients with CAD (with comparison to healthy controls), (2) the difference in serum/plasma calprotectin levels between different presentations of CAD (ACS or non‐ACS), (3) the relationship between serum/plasma calprotectin levels and outcomes following CAD, or (4) the diagnostic utility of serum/plasma calprotectin in CAD. Non‐English abstracts, review articles, and congress abstracts were excluded.

### 2.4 Screening

As a first step, duplicate studies were removed using citation manager software (EndNote version 20). Next, two reviewers independently used titles and abstracts to identify studies that met the inclusion criteria, and full texts were used to evaluate the studies for specific inclusion criteria. Any disagreements were resolved via conversation with a third reviewer. Finally, reference lists of included studies were manually searched to identify overlooked studies to be considered for inclusion in this review.

### 2.5 Data Extraction

Data extraction was performed by two independent reviewers using a predesigned spreadsheet. The following data were extracted from each study: study name (first author's name), year (publication and conduct years), population characteristics, sample size, mean age, male sex percentage, mean BMI, type of CAD (ACS or non‐ACS), calprotectin levels in each study group, area under the curve (AUC), outcome, and main findings.

### 2.6 Risk of Bias Assessment

The Newcastle−Ottawa scale (NOS) [25] was used for quality assessment using three domains: comparability, selection, and outcome. Low‐quality studies, defined as having an NOS score of less than three, were excluded from quantitative synthesis.

### 2.7 Statistical Methods

The mean and standard deviation were calculated when the median and interquartile ranges (IQRs) were reported using the method of Luo et al. [26] and Wan et al. [27]. STATA version 17 was used for statistical analyses and visualizations. Meta‐analyses comparing CAD with controls, ACS with stable CAD, ACS with control, and stable CAD with controls were performed using the random effects model (inverse variance method), which provided a standardized mean difference (SMD) and corresponding 95% confidence interval (CI). *I* ^2^ statistic was used for evaluating heterogeneity in all meta‐analyses. Publication bias was assessed with a visual assessment of the funnel plots and statistical tests of Begg's and Egger's [28, 29]. A *p* value of less than 0.05 and an *I* ^2^ value greater than 50% were defined as statistically significant results.

## 3 Results

### 3.1 Literature Search and Study Characteristics

**Figure 1:**
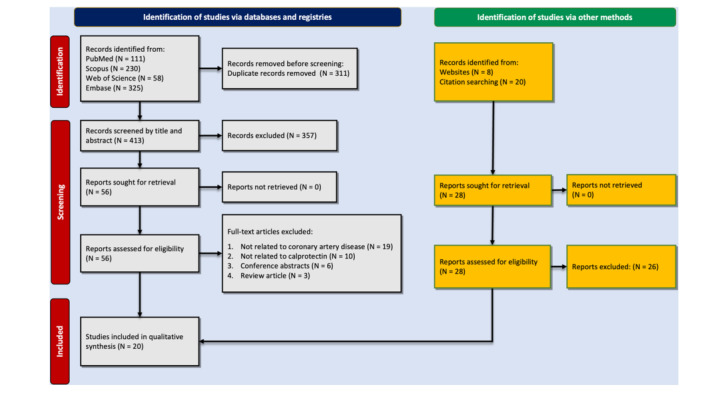
PRISMA flowchart showing the study selection process.

The initial search yielded 724 results, including 111 from PubMed, 230 from Scopus, 58 from the Web of Science, and 325 from Embase. From these, 311 were duplicates, and after screening of unique reports based on title/abstract, 56 studies remained. These went through full‐text screening for data quality so that 20 high‐quality studies were included in our systematic review [14, 23, 30, 31, 32, 33, 34, 35, 36, 37, 38, 39, 40, 41, 42, 43, 44, 45, 46, 47]. Figure 1 depicts the search details, reasons for exclusion, and a schematic diagram of the overall selection process.

The details of the included studies and their main findings are shown in Table 1. Studies were mostly conducted in China, comprising nine of the 20 studies [32, 35, 38, 40, 42, 43, 44, 45, 46]. The included studies were published between 2006 and 2022. A total of 4530 individuals were investigated in these studies, among which 3300 had CAD and 1230 were healthy controls. Thirteen studies evaluated calprotectin levels in serum [14, 23, 30, 31, 32, 34, 35, 37, 38, 42, 43, 44, 46], while six studies used plasma samples [33, 36, 39, 40, 45, 47], and one of the studies evaluated both serum and plasma levels [41]. Details of the quality assessment results of these 20 studies are shown in Supporting Information S1: Table 2. All these studies had high quality based on NOS criteria.

**Table 1:** Characteristics of studies evaluating calprotectin levels in patients with CAD.

Author	Year	Design	Location	Population	Sample size	Age	% Male	Main findings
Altwegg et al.	2007	Cross‐sectional	Switzerland	ACS patients stratified by angiographic findings and healthy controls	Total: 75 ACS: 39 SAP: 22 Control: 14	ACS: 58.4 ± 12.0 SA: 66.1 ± 12.4 Control: 60.4 ± 13.9	ACS: 77 SA: 77 HC: 71	In ACS, local (serum) MRP8/14 levels (22.0 [16.2−41.5] mg/L) were increased when compared with systemic levels [13.4 (8.1−14.7) mg/L, *p* = 0.03]. Systemic levels of MRP8/14 were markedly elevated [15.1 (12.1−21.8) mg/L, *p* = 0.001] in ACS when compared with stable CAD (4.6 [3.5−7.1] mg/L) or normal controls (4.8 [4.0−6.3] mg/L). Using a cut‐off level of 8 mg/L, MRP8/14 but not myoglobin or troponin, identified ACS presenting within 3 h from symptom onset.
Baumann et al.	2011	Cross‐sectional	Germany	CAD suspected males undergoing elective angiography	Total: 240 CAD: 166 Control: 74	CAD: 62.2 ± 9.5 Control: 58.9 ± 11.1	100%	Serum MRP8 ⁄14 was neither associated with any other cardiovascular disease risk factor nor did serum levels differ between patients with stable CAD [0.82 (0.55−1.14) lg mL) 1] and control subjects [0.91 (0.63−1.30) lg mL) 1]; *p* = 0.69). Atherosclerotic wall irregularities did not demonstrate any association with circulating MRP8 ⁄14.
Bormann et al.	2020	Prospective cohort	Germany	Patients with a final diagnosis of NSTEMI enrolling from 2011 to 2016	Total: 254 T1MI: 199 T2MI: 55	T1MI: 72.22 T2MI: 73.97	T1MI: 67.74 T2MI: 63.64	Median baseline serum MRP‐8/14 levels were higher in T2MI (*n* = 55; 3.37 [1.88−6.48] μg/mL) than in T1MI (*n* = 199; 2.4 [1.4−3.79] μg/mL) (*p* = 0.013) patients, in contrast to hs‐cTnI (T2MI: 52 [11.65−321.4] ng/L vs. T1MI: 436.5 [61.25−1973.8] ng/L; p < 0.001). T2MI is associated with higher MRP‐8/14 and lower hs‐cTnI concentrations than T1MI.
Chen et al.	2018	Case‐control	China	Patients with ACS (aged 60−92 years), CHD (aged 60−90 years), and healthy controls (aged 60−90 years)	Total: 303 ACS: 110 CHD: 110 Control: 83	ACS: 67.02 ± 12.13 CHD: 64.50 ± 8.10 Control: 67.46 ± 7.71	ACS: 54.5 CHD: 50 HC: 53.8	Serum S100A8/A9 levels were positively correlated with TLR‐4 (*r* = 0.754, *p* = 0.022) and COX‐2 levels (*r* = 0.602, *p* = 0.036) in the ACS group and with TLR‐4 (*r* = 0.586, *p* = 0.045) in the CHD group. There was no obvious correlation between S100A8/A9 and COX‐2 levels in the CHD patients (*r* = 0.078, *p* = 0.171). Peripheral blood S100A8/A9 levels were associated with ACS and also with CHD.
Healy et al.	2006	Cohort and prospective case‐control	United States	Patients undergoing coronary angiography with CAD and STEMI/cohort of apparently healthy women ≥ 45 years followed up for cardiovascular event	Total: 60 STEMI: 16 SAP: 44 Case: 255 Control: 255	STEMI: 55.3 ± 15.5 SA: 63.7 ± 10.6 Case: 61.0 ± 8.7 Control: 61.0 ± 8.7	STEMI: 75 SA: 72.7 Case: 0 Control: 0	Plasma levels of MRP‐8/14 heterodimer were higher in STEMI patients in compare with stable CAD. The risk of a first cardiovascular event increased with each increasing quartile of baseline MRP‐8/14 (range of follow‐up 0.01−5.9 years) (111 myocardial infarctions, 111 strokes, and 33 cardiovascular deaths). Risks were independent of standard risk factors and C‐reactive protein.
Jensen et al.	2010	Cohort	Denmark	pPCI‐treated STEMI patients	Total: 183 STEMI dead in follow‐up: 13 STEMI alive in follow‐up: 128 Control: 42	STEMI dead in follow‐up: 68.8 ± 13.4 STEMI alive in follow‐up: 68.6 ± 15.5 Control: 52.0 ± 12.9	STEMI dead in follow‐up: 53.4 STEMI alive in follow‐up: 68.6 Control: 76.2	The plasma calprotectin levels were significantly higher in the STEMI patients compared with the 42 healthy controls (*p* < 0.001). Plasma calprotectin levels were higher in the 13 STEMI patients who died after a median follow‐up period of 12 months compared to the STEMI patients who survived: 209 versus 174 μg/L (*p* < 0.001). In a multivariate Cox proportional hazards regression analysis, the relative risk of mortality was 1.26 per 10 μg/L increase in calprotectin. For patients with plasma calprotectin > 177 μg/L, the relative risk of mortality was 11.11.
Katashima et al.	2010	Cohort	Japan	Patients with acute MI or UAP	Total: 71 Acute MI: 55 UAP: 16	Acute MI: 66.8 ± 1.4 UAP: 74.8 ± 1.8	Acute MI: 67.27 UAP: 62.5	Serum S100A8/A9 levels on the first day were 1118 ± 115 (SE) ng/mL in AMI patients as compared with 787 ± 147 ng/mL in UAP patients. On Days 3–5, serum S100A8/A9 levels in AMI patients reached a peak value and were significantly higher than the values in UAP patients (1690 ± 144 vs. 844 ± 100 ng/mL; *p* < 0.0001).
Li et al.	2019	Prospective cohort	China	Patients with STEMI, with and without MACE	Total: 210 MACE: 24 Non‐MACE: 186	MACE: 61.5 (54.8−66.5) Non‐MACE: 59.0 (50.3−66.0)	MACE: 75 Non‐MACE: 82.8	Serum S100A8/A9 at admission was significantly higher in patients with AMI compared to controls. Moreover, patients with MACE had higher serum S100A8/A9 levels.
Marinković et al.	2019	Prospective cohort	Sweden	Patients with ACS	Total: 524 ACS: 524	NR	NR	High plasma levels of S100A8/A9 in patients with ACS are associated with lower LVEF and a higher rate of hospitalization for HF in 1‐year follow‐up.
Miyamoto et al.	2008	Cohort	Japan	Patients with SAP and UAP and age and sex‐matched healthy controls	Total: 130 SAP: 39 UAP: 53 Control: 38	SAP: 61 ± 11 UAP: 64 ± 13	SAP: 69.23 UAP: 75.47	Serum S100A8/A9 complex levels in patients with UA were significantly higher than SA and control subjects (UA 3.25 [3.08] mg/mL; SA 0.77 [0.31] mg/mL; controls 0.27 [0.11] µg/mL). There were no statistically significant differences in serum S100A8/A9 complex levels between classes II and III of UA (class II 2.6 [1.9] mg/mL; class III 4.1 [4.0] mg/mL). There were no correlations between the serum S100A8/A9 complex levels and each cardiovascular risk factor.
Peng et al.	2011	Cross‐sectional	China	T2DM patients with and without CAD	Total: 375 CAD: 170 No CAD: 205	CAD: 68 + 10 No CAD: 64 ± 10	CAD: 52.9 No CAD: 63.4	Serum MRP8/14 was higher in patients with CAD, compared with the non‐CAD group (9.7 ± 3.6 vs. 8.2 ± 3.0 ug/mL, *p* < 0.001). Also, its levels were associated with the severity of CAD.
Santilli et al.	2014	Cross‐sectional	Italy	Patients referred for elective coronary angiography due to suspected or proved IHD (chronic stable)/patients with ACS (STEMI, NSTE‐ACS)	Total: 131 Chronic IHD: 68 ACS: 63	Chronic IHD: 69 (63−77) ACS: 66.5 (61−73.5)	Chronic IHD: 47 ACS: 82.5	There was no significant difference between plasma levels of calprotectin in patients with ACS and those with chronic IHD (median [Q1−Q3] 0.93 [0.32−1.76] vs. 0.84 [0.41−1.56], *p* = 0.668)
Schaub et al.	2012	Prospective cohort	Switzerland	Consecutive patients presented to the emergency department with symptoms suggestive of AMI, such as acute chest pain and angina pectoris	Total: 398 AMI: 76 No AMI: 322	Total: 64 (51−76) AMI: 73 (63−81) No AMI: 62 (49−75)	Total: 66 AMI: 72 No AMI: 64	Serum MRP8/14 concentrations were significantly higher in patients with AMI, compared with non‐AMI cases (4.9 [3.4−7.3] vs. 3.9 [2.7−5.3] mg/L, *p* < 0.001). The AUC of MRP8/14 for diagnosis of AMI was 0.645 (95% CI 0.595−0.692). Finally, patients deceased had significantly higher levels of MRP8/14 compared to survivors (6.0 [4.0−10.2] vs. 3.9 [2.8–5.4] mg/L, *p* < 0.001).
Song et al.	2020	Prospective cohort	China	Patients with ACS who had undergone PCI were recruited consecutively from 2017 to 2018, aged from 30 to 88 years	Total: 176 Plasma calprotectin < 3681 ng/mL: 83 Plasma calprotectin > 3681 ng/mL: 93	Plasma calprotectin < 3681: 63 ± 10 Plasma calprotectin > 3681 ng/mL: 65 ± 12	Plasma calprotectin < 3681 ng/mL: 56.1 Plasma calprotectin > 3681 ng/mL: 76.1	ACS patients with no‐reflow had higher levels of plasma calprotectin compared with those without no‐reflow (6062.9 ± 999.8 vs. 3625.7 ± 1526.8 ng/mL, *p* < 0.001). The AUC of calprotectin for prediction of no‐reflow was 0.898, with sensitivity and specificity of 0.95 and 0.77 at a cut‐off value of 4748.77 ng/mL
Vora et al.	2012	Prospective cohort	United States	Patients with nontraumatic chest pain suspicious of ACS (≥ 18 years and onset of symptoms within previous 24 h)	Total: 412 Serum MRP8/14 > 3 μg/mL: 90 Serum MRP8/14 < 3 μg/mL: 321	NR	Total: 62 Serum MRP8/14 > 3 μg/mL: 67 Serum MRP8/14 < 3 μg/mL: 61	Patients presented with MI had significantly higher peak serum/plasma concentrations of MRP8/14 (3.44 vs. 2.00 μg/mL for noncardiac, *p* < 0.001). The AUC for diagnosis of MI was 0.55 (95% CI 0.51−0.60). Also, the AUC of MRP8/14 for diagnosis of ACS was 0.57 (95% CI 0.51−0.63).
Wang et al.	2019	Prospective cohort	China	Diabetic patients undergoing PCI for primary ACS	Total: 273 Serum calprotectin < 4.1 μg/mL: 122 Serum calprotectin ≥ 4.1 μg/mL: 151	Total: 63.4 ± 8.5 Serum Calprotectin < 4.1 μg/mL: 59.5 ± 7.2 Serum calprotectin ≥ 4.1 μg/mL: 61.2 ± 7.6	Total: 62.4 Serum calprotectin < 4.1 μg/mL: 60.7 Serum calprotectin ≥ 4.1 μg/mL: 62.9	The AUC of serum calprotectin for predicting MACE in diabetic patients undergoing PCI for ACS was 0.79 (95% CI 0.63−0.97), and the optimal cutoff value of 3.8 μg/mL.
Wang et al.	2020	Prospective cohort	China	Patients undergoing PCI with AMI and very late stent thrombosis (VLST), healthy controls who underwent health checkups	Total: 112 AMI: 56 VLST: 56	AMI: 59.57 + 10.65 VLST: 59.07 + 10.45	AMI: 83.93 VLST: 83.93	The mean level of serum S100A8/A9 was 3754.4 + 1688.9 ng/mL during index PCI and increased to 5517.8 + 2650.9 ng/mL at the time of VLST; in the AMI group, S100A8/A9 level was 2434.9 + 1243.4 ng/mL during index PCI and decreased to 1568.2 + 772.1 ng/mL during follow‐up, similar to that detected in the control group (1618.2 + 641.4 ng/mL).
Xia et al.	2016	Cross‐sectional	China	Patients with stable angina pectoris (SAP), unstable angina pectoris (UAP), AMI, and healthy controls	Total: 178 SAP: 46 UAP: 54 AMI: 42 Control: 36	SAP: 73.13 ± 8.25 UAP: 73.24 ± 9.06 AMI: 66.85 ± 11.52 Control: 62.00 ± 10.93	SAP: 76.1 UAP: 74.1 AMI: 76.2 Control: 72.2	Patients with CAD (UAP and AMI) had higher serum MRP8/14, compared to controls (*p* < 0.05). Also, patients with AMI group had significantly higher levels compared with UAP, SAP, and control groups (*p* < 0.05). UAP group had higher levels than SAP (36.73 ± 5.34 vs. 29.12 ± 4.57 pg/mL, *p* < 0.05).
Yu et al.	2022	Prospective cohort	China	Clinically suspected individuals for CAD or diagnosed with STEMI/NSTEMI	Total: 202 STEMI: 59 NSTEMI: 63 Control: 80	STEMI: 61.47 ± 13.16 NSTEMI: 63.25 ± 12.22 Control: 56.09 ± 10.87	STEMI: 72.9 NSTEMI: 58.7 Control: 38.7	Plasma S100A8/A9 was significantly higher in the STEMI group, compared with NSTEMI and controls (27.31 ± 7.10 vs. 21.48 ± 6.82 vs. 8.48 ± 5.69 ng/mL, *p* < 0.05).
Zhang et al.	2020	Prospective cohort	China	Diabetic patients (≥ 70 years of age) undergoing PCI for ACS	Total: 223 Serum calprotectin < 4.2 μg/mL: 97 Serum calprotectin ≥ 4.2 μg/mL: 126	Serum calprotectin < 4.2 μg/mL: 78.4 ± 3.2 Serum calprotectin ≥ 4.2 μg/mL: 78.8 ± 4.3	Serum calprotectin < 4.2 μg/mL: 52.6 Serum calprotectin ≥ 4.2 μg/mL:55.6	Serum calprotectin had an AUC of 0.79 (95% CI 0.63−0.97) in predicting MACE and an optimal cutoff value of 3.8 μg/mL.

*Note:* Data are presented as mean ± standard deviation, median (interquartile range), or percentage.

Abbreviations: ACS, acute coronary syndrome; AMI, acute myocardial infarction; AUC, area under the receiver operating characteristic curve; CAD, coronary artery disease; CHD, coronary heart disease; CI, confidence interval; HF, heart failure; IHD, ischemic heart disease; LVEF, left ventricular ejection fraction; NR, not reported; NSTEMI, non‐ST‐elevation myocardial infarction; PCI, percutaneous coronary intervention; SAP, stable angina pectoris; STEMI, ST‐elevation myocardial infarction; T2DM, type 2 diabetic patients; UAP, unstable angina pectoris; VLST, very late stent thrombosis.

### 3.2 Calprotectin Levels in Patients With CAD Versus Healthy Controls

**Figure 2:**
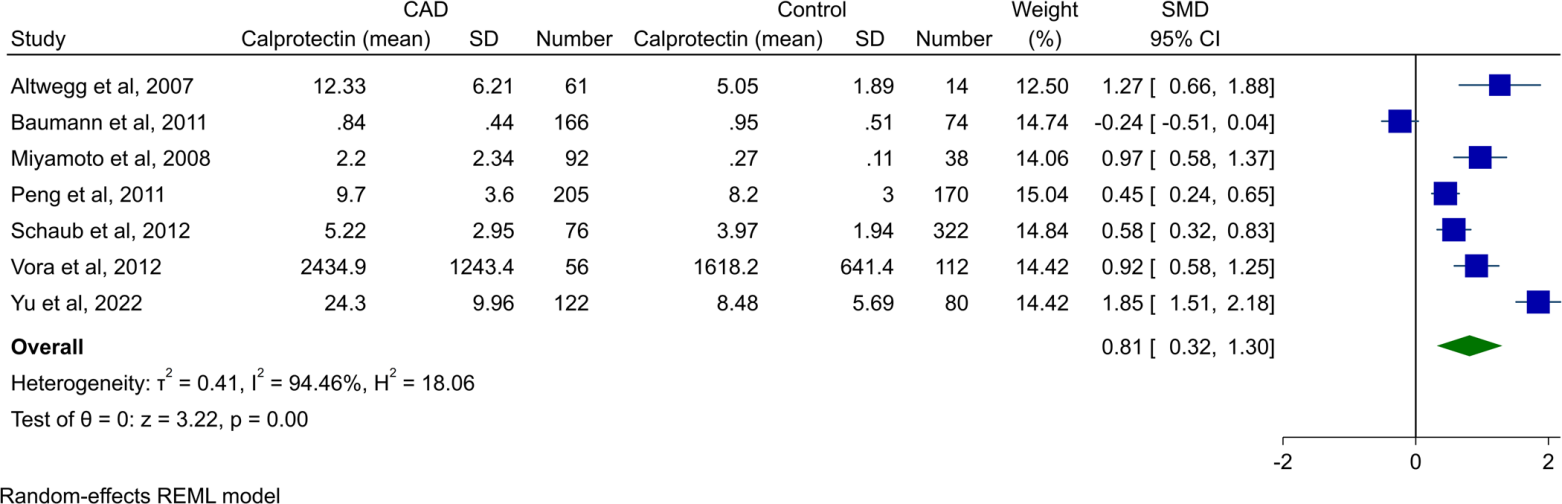
Forest plot for meta‐analysis of calprotectin levels in patients with CAD versus healthy controls.

Twenty studies assessed blood calprotectin levels in patients with CAD compared to healthy controls. Random‐effect meta‐analysis comparing serum calprotectin levels (except for Yu et al. [45], which detected plasma levels of calprotectin) in patients with CAD versus healthy controls was performed. There were 778 patients in the CAD group and 810 in the control group. As shown as a forest plot in Figure 2, patients with CAD had significantly higher calprotectin (SMD 0.81, 95% CI 0.32−1.30, *p* < 0.01). However, this analysis was associated with high heterogeneity (*I* ^2^: 94.46%).

Assessment of publication bias of this analysis showed no apparent asymmetry in the funnel plot (Supporting Information S1: Figure 1). Additionally, neither Egger's (*p* = 0.2) nor Begg's (*p* = 0.13) statistical tests demonstrated significant publication bias.

### 3.3 Calprotectin Levels in Patients With ACS Versus Stable CAD Versus Healthy Controls

**Figure 3:**
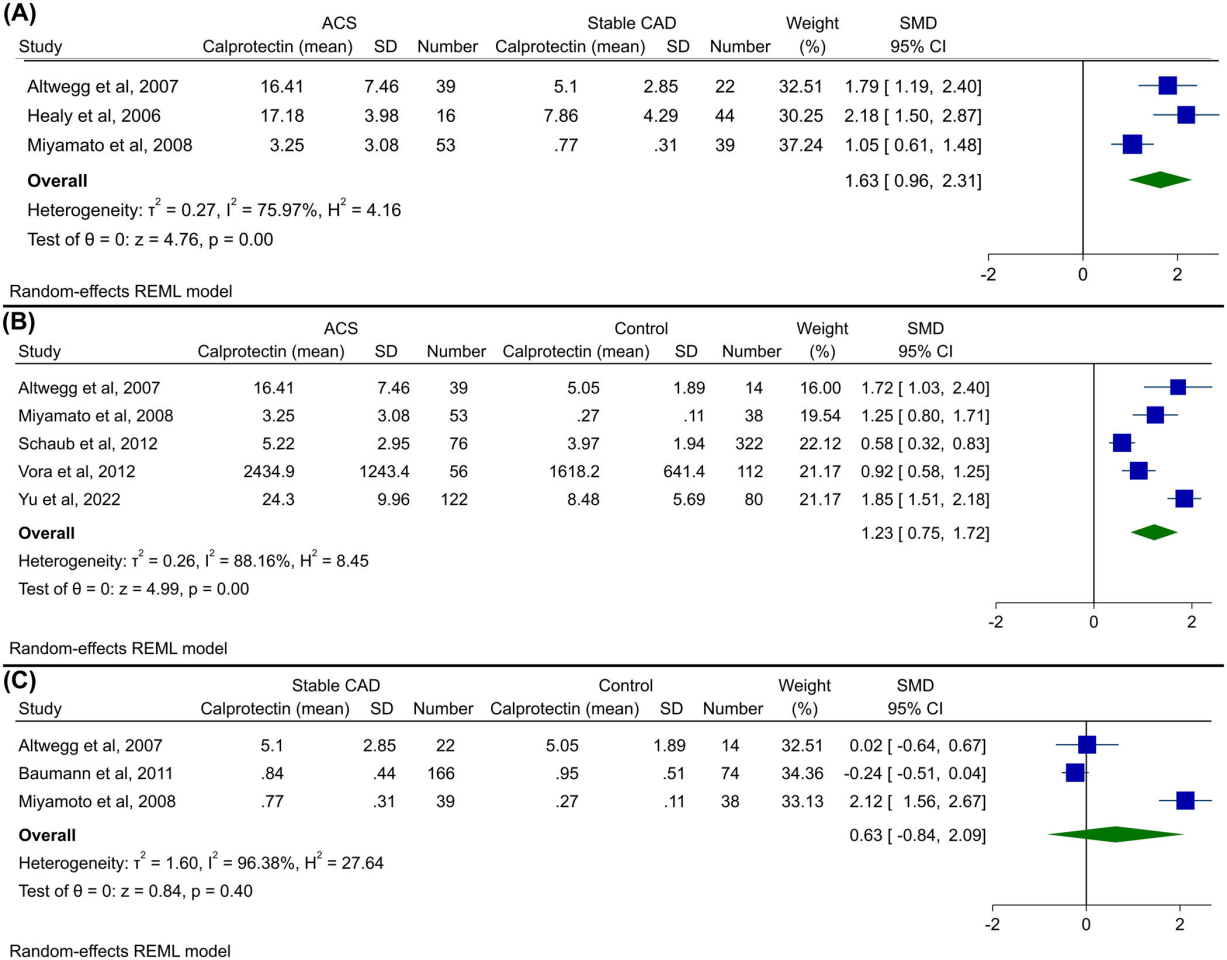
Forest plot for meta‐analysis of calprotectin levels in (A) patients with ACS versus stable CAD, (B) patients with ACS versus healthy controls, and (C) patients with stable CAD versus healthy controls.

With regard to ACS and stable CAD, meta‐analysis was also performed to compare these with healthy controls, and forest plots are shown in Figure 3. Analysis showed that patients with ACS had significantly higher levels of serum/plasma calprotectin compared to those with stable CAD (SMD 1.63, 95% CI 0.96−2.31, Figure 3A), and patients with ACS also had higher levels compared to healthy controls (SMD 1.23, 95% CI 0.75−1.72, *p* < 0.01, Figure 3B). Finally, the meta‐analysis revealed that there was no significant difference in calprotectin levels between stable CAD patients and healthy controls (SMD 0.63, 95% CI −0.84 to 2.09, *p* = 0.40, Figure 3C).

Funnel plots for these three analyses are shown in Supporting Information S1: Figures 2–4. No significant asymmetry was observed in these plots. Similarly, Begg's and Egger's tests did not show publication bias, with the exception that Egger's test was significant in the meta‐analysis of ACS versus stable CAD (ACS vs. stable CAD: Begg's *p* = 0.30, Egger's *p* < 0.01; ACS vs. healthy controls: Begg's *p* = 0.46, Egger's *p* = 0.23; stable CAD vs. healthy controls: Begg's *p* = 1.00, Egger's *p* = 0.69).

### 3.4 Calprotectin as a Diagnostic Biomarker

Altwegg et al. [30] assessed the ability of serum calprotectin to distinguish between ACS and stable CAD/controls. With a cut‐off of 8.0 mg/L, calprotectin could discriminate between these groups with sensitivity, specificity, and AUC of 95%, 92%, and 0.97, respectively. Schaub et al. [14] found an AUC of 0.645 (95% CI 0.595−0.692) for the serum calprotectin level in the diagnosis of acute MI. A combination of calprotectin level with cardiac troponin T (cTnT) and high‐sensitive (hs)‐cTnT led to AUCs of 0.907 (95% CI 0.874−0.933) and 0.950 (95% CI 0.924−0.970), respectively. Vora et al. [41] also found an AUC of 0.55 (95% CI 0.51−0.60) for the diagnosis of MI, while Yu et al. [45] reported an AUC of 0.805 (95% CI 0.744−0.866) for diagnostic efficacy of serum and plasma calprotectin in the diagnosis of MI, respectively.

Bormann et al. [31], investigated the ability of serum calprotectin level to discriminate between type 1 MI (acute MI with obstructed coronary arteries) and type 2 MI (myocardial necrosis caused by myocardial oxygen supply/demand mismatch) and reported an AUC of 0.621 (95% CI 0.537−0.705).

### 3.5 Calprotectin as a Predictor of Major Adverse Cardiovascular Events (MACE)

Two studies examined the predictive ability of serum calprotectin for MACE in patients with ACS in the setting of diabetes who were undergoing percutaneous coronary intervention (PCI) [42, 46]. Both studies reported an AUC of 0.79 for the prediction of MACE. In another study, calprotectin had a high AUC of 0.898 for the prediction of no‐reflow phenomenon in patients with ACS undergoing PCI [40].

Two studies assessed the relationship between circulating calprotectin and MACE in patients with acute MI [35, 36]. Li et al. [35] found that patients with MACE had significantly higher levels of calprotectin than controls. Similarly, Marinković et al. [36] reported that higher plasma levels of calprotectin were associated with reduced left ventricular ejection fraction and a higher rate of heart failure‐related hospitalization within 1 year.

### 3.6 Calprotectin as a Predictor of Mortality

Jensen et al. [47] investigated patients with STEMI undergoing primary PCI. They found that patients who died had significantly higher levels of plasma calprotectin compared with survivors (207 [95% CI 181–233] vs. 174 μg/L [95% CI 168–180], *p* < 0.001). Additionally, mortality was increased by 26% (HR 1.26, 95% CI 1.1–1.4, *p* = 0.001) with each 10 μg/L increase in plasma calprotectin in the adjusted model. The ROC‐AUC was 0.73 for predicting mortality.

## 4 Discussion

In the current study, we systematically reviewed the existing literature on the association of circulating calprotectin with CAD and the evaluation of its utility as a diagnostic or prognostic biomarker for ACS patients. Our meta‐analysis indicates that individuals with CAD have elevated calprotectin compared to healthy controls. Moreover, patients with ACS had significantly higher levels of calprotectin than those with stable CAD, as well as healthy controls. Increased serum calprotectin in ACS was associated with adverse prognosis and MACE.

Clinical manifestations of ACS include chest discomfort/pain, dyspnea, epigastric discomfort, nausea, fatigue, and so forth. Given the likelihood of nonspecific symptoms of ACS, especially in women, diabetics, and the elderly, along with noncardiac conditions mimicking this condition, including musculoskeletal pain, pulmonary embolism, and anxiety, the diagnosis and management of ACS remain challenging [48, 49, 50].

Clinical presentation, ECG, angiography, and cardiac biomarkers, including troponin and CK‐MB enzymes, form the diagnostic and prognostic cornerstone of ACS [51, 52]. Troponin is a component of the contractile apparatus of myocardial cells that is released in the bloodstream after myocardial necrosis and is used as a biomarker of MI [53]. With the advent of highly sensitive troponin assays, which report concentrations in nanograms per liter, other more subtle causes of myocardial injury, such as myocarditis and heart failure, will be detected and lead to positive results, thereby reducing the clinical specificity of troponin as a biomarker for MI [49, 53, 54]. Further, since troponin is not implicated in the pathogenesis of atherosclerosis, its elevation cannot be used as an indicator for determining the underlying mechanism responsible for myocardial injury: ischemic versus nonischemic [55]. Another major limitation of the troponin assay is its delayed elevation after the onset of myocardial necrosis, which typically takes 2−4 h to observe troponin elevation in blood and reaches peak levels at 24 h [52]. Overall, elevated troponin implies a diagnosis of MI, but an ideal ACS biomarker would identify high‐risk patients at early stages where preventive measures can alter the course of the disease. Due to the fact that ACS is caused by plaque instability and rupture, biomarkers of plaque inflammation may prove more effective as diagnostic tools and provide better prognostic information [14].

Inflammatory conditions such as atherosclerosis (and resulting myocardial infarction) trigger heightened expression of calprotectin. This protein is produced primarily in immune cells (specifically neutrophils and monocytes) as well as platelets and has significant intracellular and extracellular impacts [16, 17, 33]. As an intracellular signal, calprotectin governs the arrangement of microtubules and contributes to calcium‐dependent migratory responses of neutrophils and mononuclear phagocytes [56, 57]. Extracellular calprotectin primarily derives from activated or necrotic neutrophils and monocytes and serves as an innate immune mediator. Extracellular calprotectin affects Toll‐like receptor 4 (TLR4) and receptor for advanced glycation end‐products (RAGE)‐mediated signaling pathways, subsequently leading to downstream activation of nuclear factor‐kB and mitogen‐activated protein kinase (MAPK) [16, 58, 59]. The calprotectin_TLR4 interaction is implicated in the pathogenesis of ACS and results in the secretion of TNF‐α and IL‐6 [60, 61]. Calprotectin also regulates the inflammatory and regenerative stages of MI via immune cell infiltration and differentiation within the infarcted myocardium through the pathways mentioned above [62]. Specifically, TLR4 activation contributes to pathological myocardial remodeling and tissue damage after ischemic stroke [63, 64]. Also, MAPK phosphorylation exacerbates cardiomyocyte dysfunction and contributes to postischemic heart failure [65]. Importantly, short‐term inhibition of calprotectin after MI in a murine model decreased neutrophil and macrophage infiltration into the infarcted area and resulted in reduced infarction size and enhanced left ventricular ejection fraction and cardiac output [36, 65, 66, 67]. Furthermore, calprotectin can adhere to heparan sulfate proteoglycans present on the surface of vascular endothelial cells, thereby triggering endothelial activation. This activation initiates the synthesis of chemokines and increased expression of adhesion molecules, which leads to enhanced endothelial permeability to infiltrating immune cells and promotes platelet aggregation [68, 69]. Taken together, this evidence demonstrates the significant role of calprotectin in the pathogenesis of atherosclerosis and ACS in the progression of postischemic myocardial damage. Similarly, several other inflammatory biomarkers have been shown to change in CAD [70, 71].

There are two suggested sources for blood‐borne calprotectin in ACS patients. The first proposed origin is the atherosclerotic plaques [23, 30]. Examination of human carotid plaques reveals heightened calprotectin levels in vulnerable lesions, with large lipid cores, intense macrophage infiltration, low collagen, and high matrix metalloproteinases. Increased levels of calprotectin in macrophages and foam cells of unstable plaques align with macrophage recruitment patterns seen in atherosclerosis [72, 73, 74, 75, 76]. Also, clinical research affirms a correlation between levels of calprotectin and the extent of CAD, which reinforces its link to atherosclerosis. For instance, Altwegg et al. and Miyamoto et al. observed high levels of calprotectin in coronary thrombi or coronary atherosclerotic plaques in patients with ACS [30, 37], while Ionita et al. found high levels of calprotectin in rupture‐prone atherosclerotic plaques [73]. The second source of calprotectin is the infarcted myocardium [62, 63, 64]. Katashima et al. demonstrated that calprotectin is specifically expressed in neutrophils and macrophages within infarcted myocardium [34].

In the current study, we propose a classification of previous clinical studies examining calprotectin in ACS patients into three distinct categories. First, studies that investigate the levels of calprotectin in CAD patients to determine if there are any elevations in serum/plasma concentration of calprotectin in these patients (i.e., in ACS patients vs. normal, ACS patients vs. stable angina patients, etc.). In this regard, previous studies have demonstrated heightened circulating levels of calprotectin in ACS patients in comparison to stable CAD patients or normal controls [30, 39, 41] and a higher percentage of calprotectin‐positive areas in the culprit lesions of UA patients compared to those of stable angina patients, as assessed by immunohistochemical staining [37]. This goes along with the findings of five additional studies that reported increased circulating levels of calprotectin in patients after acute MI [14, 23, 33, 45, 47]. Chen et al. showed that serum calprotectin level was significantly elevated in patients with ACS and coronary heart disease compared to normal controls [32]. These findings suggest the question of whether calprotectin may be useful as a tool to distinguish ACS from stable CAD. For instance, Larsen et al. have identified that elevated levels of serum calprotectin are linked to enhanced platelet aggregation and a diminished response to aspirin in patients with CAD and diabetes [77]. Moreover, they showed that type II diabetes (itself an inflammatory condition) is an independent predictor of elevated calprotectin levels [77]. Similarly, diabetes has been shown to have associations with worse prognosis in ACS [78, 79]. Moreover, Peng et al. showed that regardless of the clinical manifestation of CAD, serum calprotectin levels were positively correlated with the severity of CAD in diabetic patients [38]. These observations underscore the importance of considering major contributors to chronic calprotectin elevation, such as other inflammatory diseases or drug history, when examining the relationship between calprotectin and ACS. Our meta‐analysis revealed a significant increase in calprotectin levels among CAD patients compared to normal controls, although this association was accompanied by high heterogeneity and some indications of publication bias. However, when comparing calprotectin levels in ACS patients to those with stable CAD or healthy controls, a significant elevation was observed in ACS patients that appeared to be without significant bias.

Second, the research on calprotectin has examined various facets of its prognostic value, such as risk stratification, its utility in assessing the prognosis of MACE before and after cardiovascular events, and the predictive value of calprotectin level in patients who underwent PCI for no‐reflow phenomenon or in very late stent thrombosis. Healy et al. conducted a nested case‐control study to assess the risk of future cardiovascular events in relation to baseline calprotectin levels in healthy women. The study found that elevated plasma levels of calprotectin independently predicted the occurrence of a first cardiovascular event within a median follow‐up period of 3 years [33]. This was in line with the findings of Cotoi et al., Schaub et al., and Marrow et al., which demonstrated that elevated calprotectin was associated with first and recurrent cardiovascular events in middle‐aged healthy individuals, with mortality after MI during a median follow‐up of 27 months for all‐cause death and with death, or recurrent myocardial infarction 30 days after an ACS, respectively [14, 80, 81]. In another study in STEMI patients with occlusion of the left anterior descending artery, plasma calprotectin levels were significantly associated with mortality with a relative risk of 1.26 per 10 μg/L increase in calprotectin in a median follow‐up of 1 year [47]. Mjelva et al. showed that in patients suspected of having ACS, elevated serum calprotectin levels were linked to a higher risk of death and recurrent MI. However, in multivariate analysis, calprotectin was not an independent predictor of future adverse events [82]. Additionally, studies have reported a significant correlation between circulating calprotectin levels and very late stent thrombosis [43], a no‐reflow phenomenon [40], and death after MI and MACE after PCI [46]. Overall, previous investigations revealed a correlation between high calprotectin levels and the first cardiovascular event in healthy individuals within cohort studies and an increased likelihood of recurrent MI and MACE in ACS patients. Thus, it is reasonable to propose that calprotectin would be a promising prognostic biomarker in ACS.

Third, studies have assessed the diagnostic utility of calprotectin levels in patients with ACS. This concept can be interpreted in various ways, such as differentiating ACS from stable angina, distinguishing NSTEMI from UA, or diagnosing ACS at earlier stages than possible by measuring troponin. In this regard, studies by Altwegg et al. and Miyamoto et al. proposed a cut‐off value of 8 mg/L and 1.2 µg/mL for serum calprotectin as an identifier of ACS presenting within 3 h from symptom onset (before troponin elevation) and a discriminative factor between patients with UA and SA for the diagnosis of UA, respectively [30, 37]. In another study, serum calprotectin was reported to be useful in discriminating the types of MI, complementing troponin [31]. However, subsequent studies revealed some discrepancies. For instance, in two cohort studies where calprotectin levels were serially measured, one reported a temporary surge in calprotectin in the serum of acute MI patients, reaching its peak between 3 and 5 days after the onset of MI [34], while the second study observed a peak in serum calprotectin levels within 4−6 h of clinical presentation in MI patients [41]. Nevertheless, overall diagnostic performance was poor and inferior to troponin in both of these investigations [34, 41]. These results align with recent reports that have confirmed the poor diagnostic accuracy of calprotectin in ACS patients and indicate ineffectiveness in predicting the extent of atherosclerotic changes [14, 23]. In general, despite some discrepancies in results that may stem from differences in basal characteristics, sample type (serum/plasma), course of the disease, or study design, it appears that calprotectin level may not be an effective diagnostic tool for identifying ACS patients. However, as mentioned in the previous study by Schaub et al. [14], the combination of calprotectin detection with troponin may enhance the diagnosis of ACS patients. Hence, further studies are recommended.

Our study findings have several clinical and research implications. Higher calprotectin levels in patients with CAD and also differences among CAD subtypes could guide clinicians in providing better care for these patients. Moreover, calprotectin measurement in patients with the disease could have prognostic utility if confirmed in further studies. These suggest that as an easy‐to‐use marker, calprotectin, could be used as a risk marker for the development of CAD in the healthy general population. As a routinely measured marker, it could have the potential to be added to the laboratory panel in patients with cardiovascular disorders. All these could be beneficiary for cardiologists as well as primary care physicians if future studies are in accordance with these findings.

This study was the first systematic review that assessed the association between systemic calprotectin levels and CAD, as well as its subtypes, including ACS and stable CAD. In addition to comparing each of these with each other and with healthy controls, we reported the prognostic significance of this biomarker and its correlation with adverse events and mortality. However, there are certain limitations to this study that should be mentioned. First, the high heterogeneity observed in all meta‐analyses could be considered a limitation and suggests further homogenous studies in this regard. Second, there were a limited number of studies that assessed the diagnostic ability of calprotectin, and hence, more studies are needed in this regard. Moreover, the calculation of mean and SD based on the median and IQR based on the methods suggested by Luo et al. [26] and Wan et al. [27], despite being used commonly, could lead to bias in the results. Finally, the low sample size in several of the included studies could threaten the generalizability of the results observed.

## 5 Conclusion

Our findings indicate that patients with ACS exhibited increased serum/plasma calprotectin levels. Although calprotectin level correlated positively with prognosis, its diagnostic value was suboptimal. Several studies have suggested a link between platelet activation and elevated calprotectin and have proposed calprotectin as a potential target for new therapeutic agents. Future investigations should explore optimal cut‐off values for predictive purposes in patients with ACS. Additionally, in these studies, serial measurement of calprotectin levels could be performed to assess its effectiveness as a biomarker to track atherosclerotic plaque progression. This may provide clinicians with another effective method for evaluating high‐risk patients and improving the management of ACS. Studies should also consider the confounding effects of anti‐ischemic drugs, such as heparin, on inflammatory status that could impact calprotectin levels.

## Author Contributions

**Tara Reshadmanesh and Amir Hossein Behnoush:** literature search, writing–original draft, conceptualization, formal analysis, visualization. **Maedeh Farajollahi**, **Amirmohammad Khalaji, and Elina Ghondaghsaz:** writing–original draft, data curation. **Hassan Ahangar:** study conception, critical revision, manuscript drafting (editing). All authors read and approved the final manuscript.

## Ethics Statement

The authors have nothing to report.

## Consent

The authors have nothing to report.

## Conflicts of Interest

The authors declare no conflicts of interest.

## Supporting information

Supporting information.

## Data Availability

Data sharing is not applicable to this article as no new data were created or analyzed in this study.
