# Supplementary material for: Circulating Levels of Calprotectin as a Biomarker in Patients With Coronary Artery Disease: A Systematic Review and Meta‐Analysis
Source: Clin Cardiol. 2024 Jul 4;47(7):e24315. doi: 10.1002/clc.24315 (PMC11222710; doi:10.1002/clc.24315)
Supplement: Supplementary file 1 — Supporting information. [file CLC-47-e24315-s001.docx]

**Supplementary Materials**

***Supplementary Table 1.*** *Search strategy for each database*

| **Query** | | **Results**  **(October 16, 2023)** |
| --- | --- | --- |
| **PubMed** | | |
| #1 | (“Coronary”[tiab] OR "Myocardial Ischemia"[Mesh] OR "Myocardial ischemia"[tiab] OR "Coronary Artery Disease"[Mesh] OR "Coronary Atherosclerosis"[tiab] OR "Coronary Disease"[Mesh] OR "Coronary Heart Disease*"[tiab] OR "ischemic heart"[tiab]) | 685,314 |
| #2 | (“Calprotectin*”[tiab] OR "Leukocyte L1 Antigen Complex"[Mesh] OR “Calcium-Binding Myeloid Protein P8,14”[tiab] OR “Calcium Binding Myeloid Protein P8,14”[tiab] OR “Calgranulin”[tiab] OR “Migratory Inhibitory Factor-Related Protein MRP”[tiab] OR “Migratory Inhibitory Factor Related Protein MRP”[tiab] OR “Myelomonocytic Antigen L1”[tiab] OR “Antigen L1, Myelomonocytic”[tiab] OR “L1 Antigen”[tiab] OR “Antigen, L1”[tiab] OR “27E10 Antigen”[tiab] OR “Antigen, 27E10”[tiab] OR “Leukocyte L1 Protein”[tiab] OR “L1 Protein, Leukocyte”[tiab]) | 7,153 |
| #3 | #1 AND #2 | 111 |
| **Scopus** | | |
| #1 | TITLE-ABS-KEY(“Coronary” OR "Myocardial Ischemia" OR "Myocardial ischemia" OR "Coronary Artery Disease" OR "Coronary Atherosclerosis" OR "Coronary Disease" OR "Coronary Heart Disease*" OR "ischemic heart") | 835,522 |
| #2 | TITLE-ABS-KEY(“Calprotectin*” OR "Leukocyte L1 Antigen Complex" OR “Calcium-Binding Myeloid Protein P8,14” OR “Calcium Binding Myeloid Protein P8,14” OR “Calgranulin” OR “Migratory Inhibitory Factor-Related Protein MRP” OR “Migratory Inhibitory Factor Related Protein MRP” OR “Myelomonocytic Antigen L1” OR “Antigen L1, Myelomonocytic” OR “L1 Antigen” OR “Antigen, L1” OR “27E10 Antigen” OR “Antigen, 27E10” OR “Leukocyte L1 Protein” OR “L1 Protein, Leukocyte”) | 13,185 |
| #3 | #1 AND #2 | 230 |
| **Web of Science** | | |
| #1 | TS=(“Coronary” OR "Myocardial Ischemia" OR "Myocardial ischemia" OR "Coronary Artery Disease" OR "Coronary Atherosclerosis" OR "Coronary Disease" OR "Coronary Heart Disease*" OR "ischemic heart") | 622,522 |
| #2 | TS=(“Calprotectin*” OR "Leukocyte L1 Antigen Complex" OR “Calcium-Binding Myeloid Protein P8,14” OR “Calcium Binding Myeloid Protein P8,14” OR “Calgranulin” OR “Migratory Inhibitory Factor-Related Protein MRP” OR “Migratory Inhibitory Factor Related Protein MRP” OR “Myelomonocytic Antigen L1” OR “Antigen L1, Myelomonocytic” OR “L1 Antigen” OR “Antigen, L1” OR “27E10 Antigen” OR “Antigen, 27E10” OR “Leukocyte L1 Protein” OR “L1 Protein, Leukocyte”) | 7,109 |
| #3 | #1 AND #2 | 58 |
| **Embase** | | |
| #1 | (“Coronary” OR "Myocardial Ischemia" OR "Myocardial ischemia" OR "Coronary Artery Disease" OR "Coronary Atherosclerosis" OR "Coronary Disease" OR "Coronary Heart Disease*" OR "ischemic heart") | 996,558 |
| #2 | (“Calprotectin*” OR "Leukocyte L1 Antigen Complex" OR “Calcium-Binding Myeloid Protein P8,14” OR “Calcium Binding Myeloid Protein P8,14” OR “Calgranulin” OR “Migratory Inhibitory Factor-Related Protein MRP” OR “Migratory Inhibitory Factor Related Protein MRP” OR “Myelomonocytic Antigen L1” OR “Antigen L1, Myelomonocytic” OR “L1 Antigen” OR “Antigen, L1” OR “27E10 Antigen” OR “Antigen, 27E10” OR “Leukocyte L1 Protein” OR “L1 Protein, Leukocyte”) | 20,373 |
| #3 | #1 AND #2 | 325 |
| **Total records** | | **724** |
| **Total records without duplicates** | | **413** |

***Supplementary Table 2.*** *Qualities of included studies based on NOS*

| **Study** | **Selection** | | | | **Comparability** | **Outcome** | | | **Overall**  **Score** |
| --- | --- | --- | --- | --- | --- | --- | --- | --- | --- |
|  | **Representation of exposed cohort** | **Selection of the non-exposed cohort** | **Ascertainment of exposure** | **Outcome of interest presence** |  | **Assessment of outcome** | **Sufficient length of follow-up** | **Loss to follow-up** |  |
| **Altwegg et al. (2007)** | * | * | * | * | - | * | *  (In-hospital) | * | 7 |
| **Baumann et al. (2011)** | * | * | * | * | - | * | *  (In-hospital) | * | 7 |
| **Bormann et al. (2020)** | * | * | * | * | - | * | *  (In-hospital) | * | 7 |
| **Chen et al. (2018)** | * | * | * | * | - | * | *  (In-hospital) | * | 7 |
| **Healy et al. (2006)** | * | * | * | * | ** | * | *  (Median 2.9 years) | * | 9 |
| **Jensen et al. (2010)** | * | * | * | * | - | * | *  (Median 12 months) | * | 7 |
| **Katashima et al. (2010)** | * | * | * | * | - | * | *  (In-hospital) | * | 7 |
| **Li et al. (2019)** | * | * | * | * | - | * | *  (2 days) | * | 7 |
| **Marinković et al. (2019)** | * | * | * | * | - | * | *  (1-year) | * | 7 |
| **Miyamoto et al. (2008)** | * | * | * | * | ** | * | *  (In-hospital) | * | 9 |
| **Peng et al. (2011)** | * | * | * | * | - | * | *  (In-hospital) | * | 7 |
| **Santilli et al. (2014)** | * | * | * | * | - | * | *  (In-hospital) | * | 7 |
| **Schaub et al. (2012)** | * | * | * | * | - | * | *  (Median 27 months) | * | 7 |
| **Song et al. (2020)** | * | * | * | * | - | * | *  (In-hospital) | * | 7 |
| **Vora et al. (2012)** | * | * | * | * | - | * | *  (In-hospital) | * | 7 |
| **Wang et al. (2019)** | * | * | * | * | - | * | *  (12 months) | * | 7 |
| **Wang et al. (2020)** | * | * | * | * | ** | * | *  (3 years) | * | 9 |
| **Xia et al. (2016)** | * | * | * | * | - | * | *  (In-hospital) | * | 7 |
| **Yu et al. (2022)** | * | * | * | * | - | * | *  (In-hospital) | * | 7 |
| **Zhang et al. (2020)** | * | * | * | * | - | * | *  (Median 12 months) | * | 7 |

*
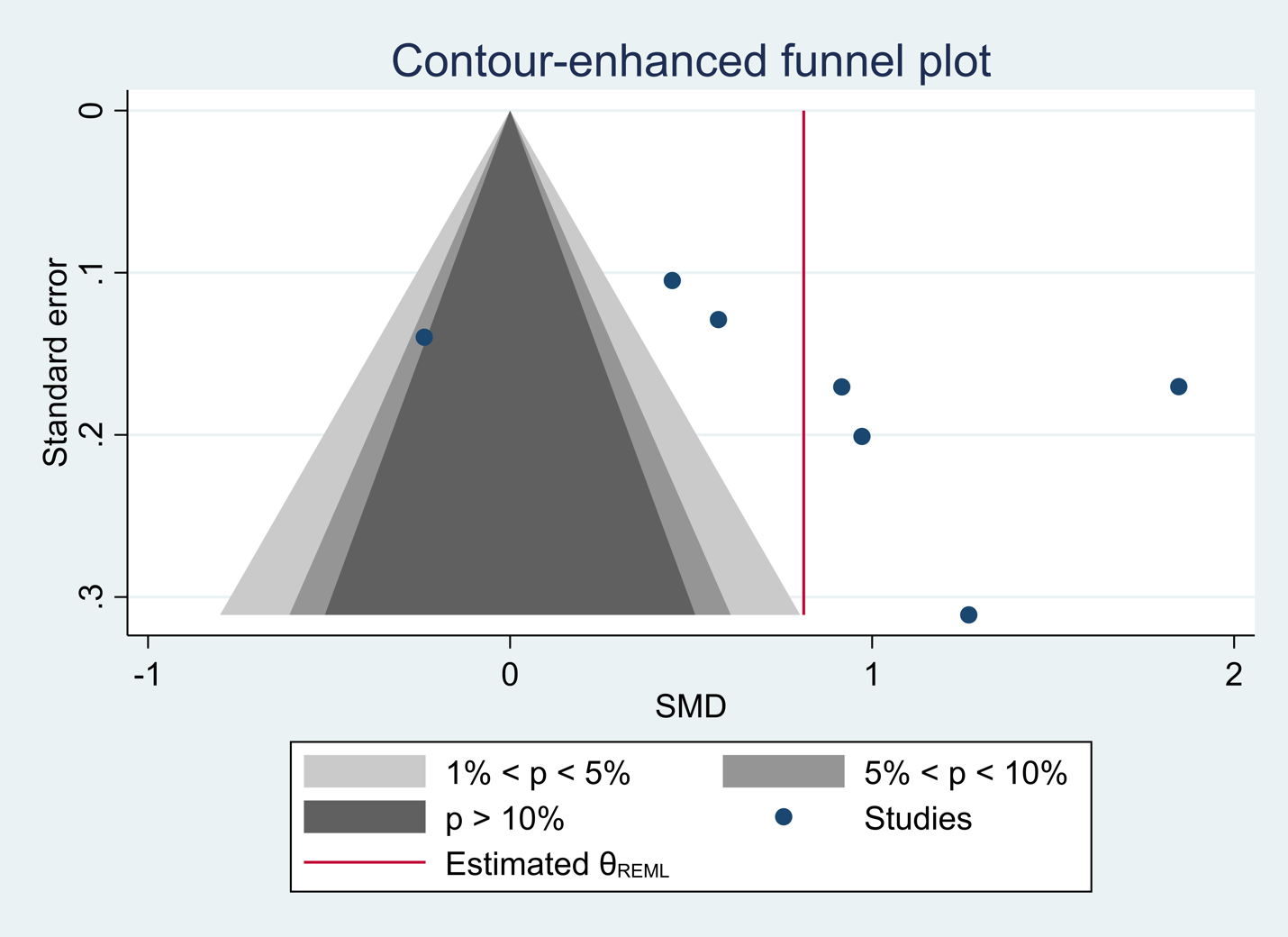
*

***Supplementary Figure 1.*** *Funnel plot for meta-analysis of calprotectin levels in patients with CAD vs. healthy controls*

*
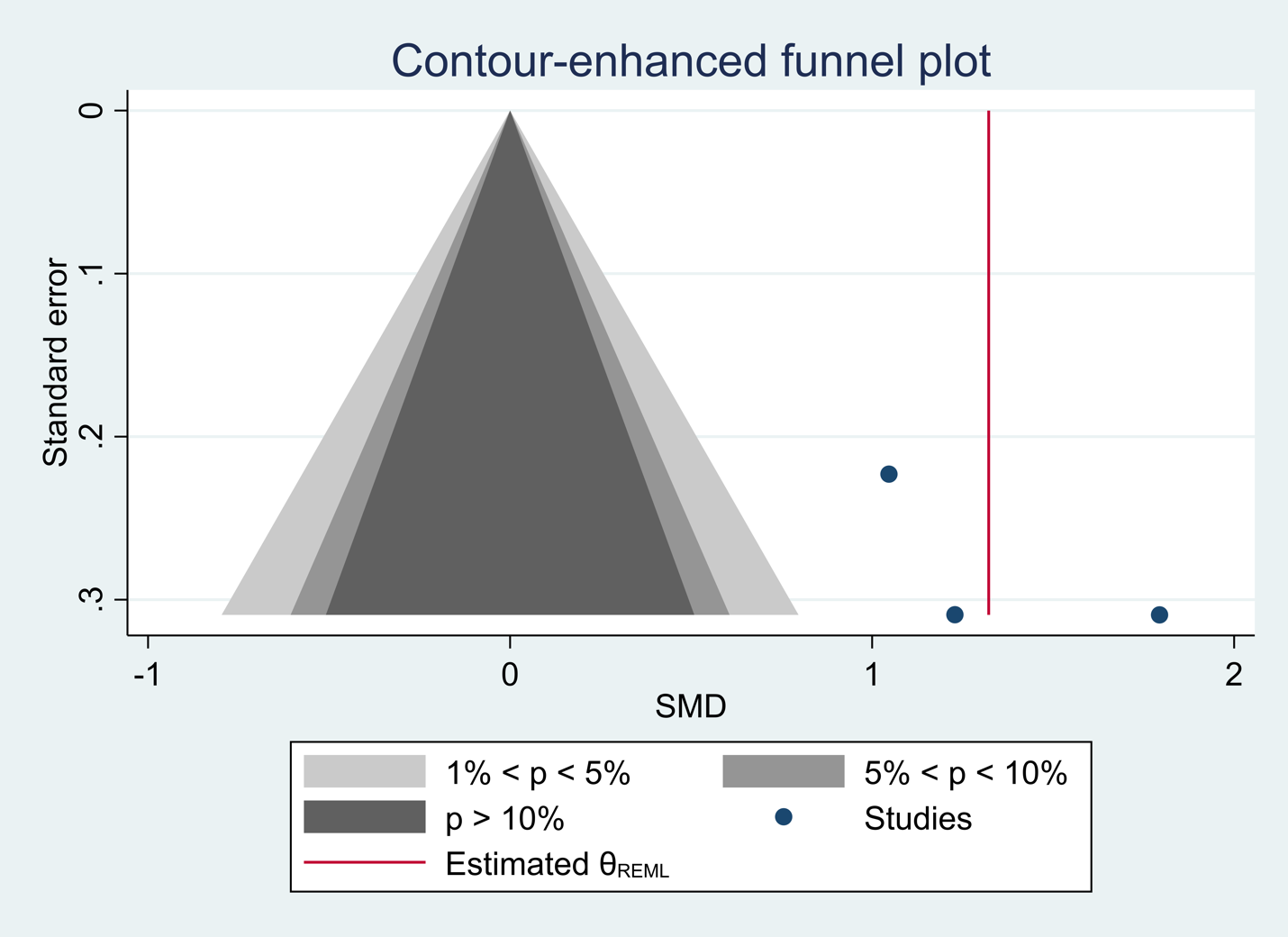
*

***Supplementary Figure 2.*** *Funnel plot for meta-analysis of calprotectin levels in patients with ACS vs. stable CAD*

*
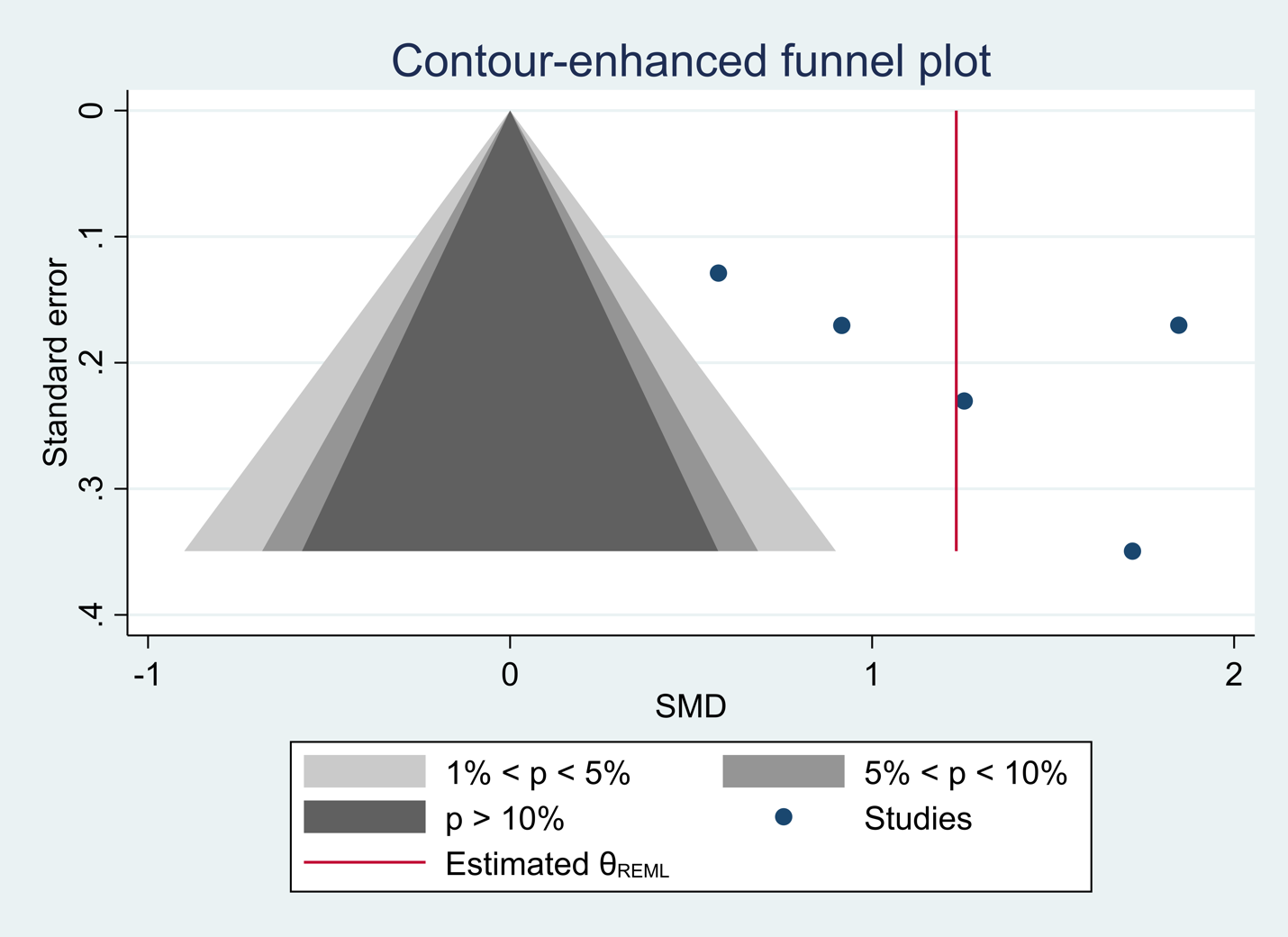
*

***Supplementary Figure 3.*** *Funnel plot for meta-analysis of calprotectin levels in patients with ACS vs. healthy controls*

*
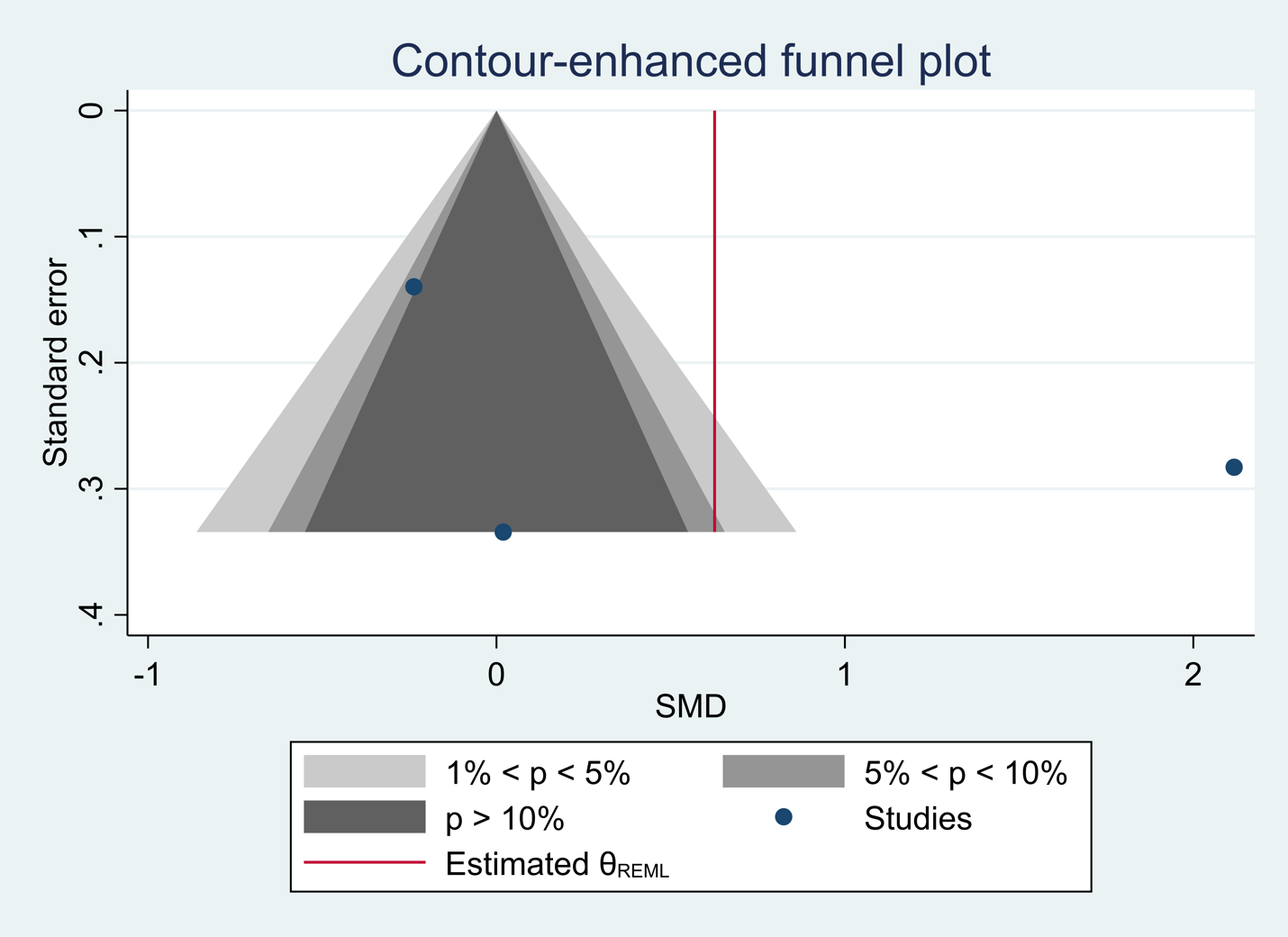
*

***Supplementary Figure 4.*** *Funnel plot for meta-analysis of calprotectin levels in patients with stable CAD vs. healthy controls*
